# Different effects of I-wave periodicity repetitive TMS on motor cortex interhemispheric interaction

**DOI:** 10.3389/fnins.2023.1079432

**Published:** 2023-06-30

**Authors:** Dongting Tian, Shin-Ichi Izumi

**Affiliations:** ^1^Department of Physical Medicine and Rehabilitation, Tohoku University Graduate School of Medicine, Sendai, Japan; ^2^Graduate School of Biomedical Engineering, Tohoku University, Sendai, Japan

**Keywords:** I-wave, neural plasticity, interhemispheric interaction, neural circuits, motor cortex, transcranial magnetic stimulation, neuromodulation

## Abstract

**Background:**

Activity of the neural circuits in the human motor cortex can be probed using transcranial magnetic stimulation (TMS). Changing TMS-induced current direction recruits different cortical neural circuits. I-wave periodicity repetitive TMS (iTMS) substantially modulates motor cortex excitability through neural plasticity, yet its effect on interhemispheric interaction remains unclear.

**Objective:**

To explore the modulation of interhemispheric interaction by iTMS applied in different current directions.

**Materials and Methods:**

Twenty right-handed healthy young volunteers (aged 27.5 ± 5.0 years) participated in this study with three visits. On each visit, iTMS in posterior–anterior/anterior–posterior direction (PA-/AP-iTMS) or sham-iTMS was applied to the right hemisphere, with corticospinal excitability and intracortical facilitation of the non-stimulated left hemisphere evaluated at four timepoints. Ipsilateral silent period was also measured at each timepoint probing interhemispheric inhibition (IHI).

**Results:**

PA- and AP-iTMS potentiated cortical excitability concurrently in the stimulated right hemisphere. Corticospinal excitability of the non-stimulated left hemisphere increased 10 min after both PA- and AP-iTMS intervention, with a decrease in short-interval intracortical facilitation (SICF) observed in AP-iTMS only. Immediately after the intervention, PA-iTMS tilted the IHI balance toward inhibiting the non-stimulated hemisphere, while AP-iTMS shifted the balance toward the opposite direction.

**Conclusions:**

Our findings provide systematic evidence on the plastic modulation of interhemispheric interaction by PA- and AP-iTMS. We show that iTMS induces an interhemispheric facilitatory effect, and that PA- and AP-iTMS differs in modulating interhemispheric inhibition.

## 1. Introduction

Inhibition and facilitation in the human motor cortex have been examined extensively using transcranial magnetic stimulation (TMS) (Valero-Cabré et al., 2017; Tian and Izumi, 2023). In epidural recordings, the TMS-evoked corticospinal descending volleys are named in arrival order as the direct wave (D-wave, with the shortest latency, reflecting the direct excitation of cortical pyramidal tract neurons, PTNs) and the indirect waves (I-waves, reflecting the indirect excitation impinging on PTNs from the non-PTN neuron circuits) (Patton and Amassian, 1954; Di Lazzaro et al., 2012; Di Lazzaro and Rothwell, 2014). The formation of I-waves has been regarded as a reflection of cortical circuit activity evoked by TMS, which involves the activity of both excitatory pyramidal neurons and inhibitory interneurons in the motor cortex (Di Lazzaro and Ziemann, 2013). Considering the properties of I-waves, one most known observation is the selectivity of I-waves by TMS-induced current direction. In particular, TMS-induced current in posterolateral-anteromedial (PA) direction preferentially recruits early I-wave, while anteromedial-posterolateral (AP) current preferentially elicits late I-waves (Day et al., 1989; Sakai et al., 1997; Di Lazzaro et al., 2001; D’Ostilio et al., 2016). However, it is possible that the “late I-waves” evoked by AP-TMS at late I-wave timings may originate from neural populations which are different from those generating late I-waves in PA stimulation [for a review see (Opie and Semmler, 2021)]. At the microscopic scale, as neuronal excitation by TMS mainly propagates along axons, reversing the current direction may result in different excitation of the anterior and posterior banks of the precentral gyrus, in which the axons descend in opposite directions (Siebner et al., 2022). Additionally, even in the same cortical column, it is possible that altering TMS induced current direction may cause excitation in different neuron populations. Given that callosal projections differ among neuron subtypes (Petreanu et al., 2007; Bakken et al., 2021), changing TMS-evoked current direction can not only result in different presence of I-waves, but may also alter the induced activity in local and remote cortical neural circuits.

Short-interval intracortical facilitation (SICF) has been considered as a reliable paired-pulse TMS paradigm probing the intracortical I-wave circuits (Van den Bos et al., 2018; Qasem et al., 2020; Ziemann, 2020). By applying a suprathreshold first pulse and a peri-threshold second pulse at a 1–6 ms interstimulus interval (ISI), facilitation peaks emerge at ISIs of 1.1–1.7, 2.3–2.9, and 4.1–4.4 ms, corresponding to the onset latency of the I1-, I2-, I3-wave in epidural recordings (Tokimura et al., 1996; Ziemann et al., 1998a,b). In a manner of repeating paired-pulse SICF, I-wave periodicity repetitive TMS (iTMS) was further proven able to modulate synaptic plasticity via long-term potentiation (LTP)-like effects (Thickbroom et al., 2006; Di Lazzaro et al., 2007; Sewerin et al., 2011; Opie et al., 2021), potentiating single-pulse MEP and paired-pulse SICF (Silbert et al., 2011; Opie et al., 2021; Sasaki et al., 2022). However, even if iTMS was devised on the basis of I-wave facilitation (Thickbroom et al., 2006), iTMS has always been studied in a relatively fixed pattern (i.e., applying iTMS in PA current direction, and assessing cortical circuit activity of the stimulated hemisphere) [for a review see (Kidgell et al., 2016)]. Whereas studies on iTMS have provided valuable evidence on the excitatory effects of iTMS, investigation on specific mechanisms such as how iTMS interacts with the intrinsic properties of I-waves, or how the selectivity of early and late I-waves affects the plastic effects, remains absent. Additionally, although the increase of SICF in the stimulated hemisphere after iTMS has been identified, the iTMS interhemispheric effects have not yet been investigated, leaving its impact on cortical interhemispheric interaction largely unknown. Although studies on excitatory quadripulse stimulation (QPS) with stimulation pattern comparable with iTMS have provided clues to a facilitatory effect on the non-stimulated hemisphere for this protocol (Di Lazzaro et al., 2010; Tsutsumi et al., 2013), direct evidence is still lacking regarding how iTMS modulates interhemispheric interaction.

In the present study, we aimed to illustrate the motor cortex circuit wiring by exploring the modulation of interhemispheric interaction by iTMS with different I-wave selectivity. We integrated I-wave selectivity in iTMS by applying iTMS with early I-wave selectivity (PA current direction and early I-wave timing, PA-iTMS) and late I-wave selectivity (AP current direction and late I-wave timing, AP-iTMS) respectively. Particularly, we firstly applied AP-iTMS as a novel protocol, which has not been reported in previous literature. Since there is no evidence showing the online effects of AP-iTMS, and that the presence of iTMS online effects would be a premise for investigating the interhemispheric effects, the present study also serves as a pilot study exploring the online effects of AP-iTMS. For the iTMS interhemispheric effects, we adopted paired-pulse SICF and long-interval intracortical facilitation (LICF) as the probe, to examine the effects of iTMS on intracortical facilitation (ICF, including SICF and LICF) in the non-stimulated hemisphere. In addition, using ipsilateral silent period [iSP, the transient EMG interruption of the contracting muscle ipsilateral to a suprathreshold TMS pulse (Hupfeld et al., 2020)] as an indicator of interhemispheric inhibition (IHI), we sought to address the specific modulation of IHI by PA- and AP-iTMS. We presumed that: (1) AP-iTMS can induce gradual concurrent MEP increase similar to PA-iTMS; (2) unilateral iTMS intervention potentiates the excitability in both hemispheres similar to the effect of excitatory QPS, which can be reflected in the change of single-pulse MEP and paired-pulse ICF; and (3) ICF and IHI modulation in the non-stimulated hemisphere differ between PA- and AP-iTMS.

## 2. Materials and methods

### 2.1. Subjects

Twenty healthy right-handed adults (aged 27.5 ± 5.0 years; 7 males) with no reported history or current signs of neurological or musculoskeletal impairment participated in the study. Prior to the experiment, handedness was assessed using the Edinburgh Handedness Inventory (Oldfield, 1971). Screening for TMS and rTMS contraindications was conducted according to the International Federation of Clinical Neurophysiology (IFCN) criteria (Rossi et al., 2011, 2021). Written informed consent was priorly obtained from all subjects. The experiment was conducted in accordance with the Declaration of Helsinki and was approved by the Ethics Committee at Tohoku University Hospital (Protocol Identification Number: 25767).

### 2.2. Equipment and configuration

#### 2.2.1. TMS equipment

The experiment was performed using two Magstim 200^2^ monophasic stimulators connected to a Bistim module (Magstim Co., Whitland, United Kingdom). To avoid coil overheating, two coils were used in the experiment. A 70 mm MAG-9925-00 figure-of-eight coil was used in TMS evaluation, and a D70 Alpha BI coil (4510–00, Magstim Co., Whitland, United Kingdom) was used for the iTMS intervention at the M1 hotspot of both hemispheres. Ice packs were used to cool the non-used coil as only one coil was used in either TMS evaluation or intervention. The M1 hotspot was determined as the point where suprathreshold TMS pulses evoked maximum MEP from the abductor pollicis brevis (APB) muscle contralateral to TMS. For PA stimulation, the coil was oriented to generate 45° PA induced electric field in the M1 target area. For AP stimulation, the coil current direction was reversed 180° from PA orientation. When applying AP- and PA-iTMS, the coil junction center was placed tangentially over the scalp at the APB hotspot marked by a felt-tip pen. During sham iTMS, the coil plane was held perpendicular to the cranial plane over M1. TMS was automatically executed using customized MATLAB scripts (MATLAB 2021a, the same hereinafter, The MathWorks, Inc., Natick, MA, United States) using the MAGIC toolbox (Habibollahi Saatlou et al., 2018). The coils were held by two mechanical arms (Manfrotto 244, VitecGroup, Italy) during the experiment, for TMS evaluation and iTMS intervention, respectively.

#### 2.2.2. EMG recording

EMG was collected from bilateral APB using disposable surface electrodes (Ambu Blue Sensor N, N-00-S/25, Ambu A/S, Ballerup, Denmark) in a lengthwise belly-belly montage, with the reference electrode attached to the ulnar styloid process (Corneal et al., 2005). Surface EMG was recorded using a MEG-6116 M bio-amplifier (Nihon-kohden, Tokyo, Japan) and a PowerLab 16/35 hardware. Recorded EMG signal was amplified 1,000×, band-pass filtered (20–450 Hz), digitized at 10 k Hz, stored, and analyzed offline using LabChart Pro 8.0 software (AD Instruments Inc.) and MATLAB 2021a.

### 2.3. Protocol

#### 2.3.1. Experiment procedure

**Figure 1:**
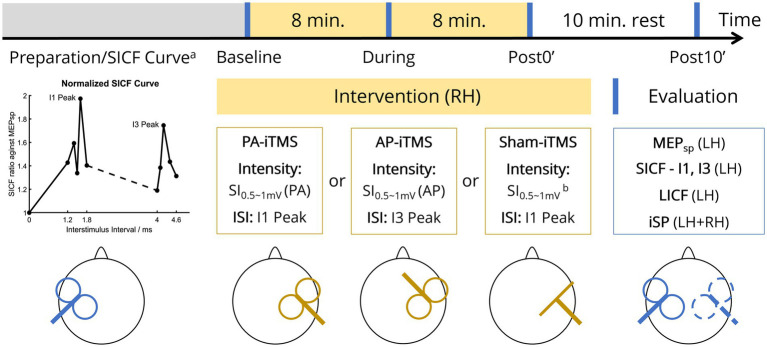
Experiment protocol. On each visit of the subject, one of the three types of iTMS intervention was performed in two sessions (yellow blocks, 16 min in total). TMS evaluation (blue vertical lines) was performed at four timepoints (before iTMS ‘Baseline’, half-time of iTMS ‘During’, immediately after iTMS ‘Post0’, and 10 minutes after iTMS ‘Post10’). Prior to the first experiment, SICF curve was measured to probe the individual I1/I3 peak of the subject (lower left panel). MEP_sp_, single-pulse MEP; LH, left hemisphere; RH, right hemisphere; rMT, resting motor threshold; SI_0.5-1mV_, single-pulse TMS intensity that evoked 0.5–1 mV MEP; PA/AP, posterolateral-anteromedial/anteromedial-posterolateral (TMS coil orientation); ISI, interstimulus interval. Superscript: ^a^SICF curve was measured at the first session only, prior to the baseline TMS evaluation; ^b^coil placed perpendicular to the scalp over the right M1 hand area.

Each subject underwent the experiment protocol on three sessions (~1.5 h each) separated by at least 24 h (inter-session interval mean ± SD: 6.8 ± 5.5 days, Figure 1). During the experiment, subjects were comfortably seated with both arms rested aside of the body. The subject was instructed to rest his/her head on the back of a high-back armchair. Padded head cushion was used to support and fixate the subject’s head. The stimulation parameters of iTMS intervention and TMS evaluation were determined prior to the experiment, including bilateral resting motor threshold (rMT), SI_0.5-1mV_ (the TMS stimulus intensity that evoked 0.5-1 mV MEP) and the SICF curve (details stated in 2.3.2 Preparation and SICF curve measurement). ITMS was delivered to the right hemisphere and TMS evaluation was performed at four timepoints (baseline, during, post0’, post10’), with single-pulse MEP (MEP_sp_), SICF at I1 and I3 peak timings (SICF-I1 and SICF-I3), LICF in the left hemisphere and bilateral iSP assessed. To attenuate random attention fluctuation (Conte et al., 2007, 2008), a printed black fixation cross of 5 × 5 cm was set 50 cm in front of the subject’s eyes. Subjects were instructed to keep fixating on the cross during the experiment.

#### 2.3.2. Preparation and SICF curve measurement

On each visit, bilateral rMT and SI_0.5-1mV_ of the subject was measured by single-pulse TMS in PA direction. The rMT was defined as the minimal TMS intensity that evoked five MEPs (peak-to-peak amplitude >50 μV) out of 10 consecutive stimuli and was recorded as a percentage of maximum stimulator output (% MSO). Considering the substantial rMT difference in PA and AP orientation (Delvendahl et al., 2014; D’Ostilio et al., 2016; Hannah and Rothwell, 2017), on the session with AP-iTMS intervention, rMT and SI_0.5-1mV_ of the right hemisphere were additionally measured in AP prior to the session.

On the first visit of the subject only, the SICF curve (the ISI-MEP curve of paired-pulse SICF measured in PA direction) of the left hemisphere was measured before the beginning of the experiment (Figure 1, lower left panel) (Qasem et al., 2020). In this measurement, 10 single-pulse stimuli with SI_0.5-1mV_ were first delivered to the left hemisphere, from which the peak-to-peak MEP amplitude was averaged. Subsequently, paired-pulse SICF with a test stimulus (TS) of SI_0.5-1mV_ followed by a conditioning stimulus (CS) of 90% rMT was delivered. Ten ISIs were tested five times in random order to detect the ISI peak of I1-wave (1.2, 1.4, 1.5, 1.6, 1.8 ms) and I3-wave (4.0, 4.1, 4.2, 4.4, 4.6 ms) (Opie et al., 2018; Qasem et al., 2020). At each ISI, paired-pulse MEP amplitude was averaged and then normalized to (the averaged) “MEP_sp_” (amplitude). As little hemispheric asymmetry exists in SICF (Ilic et al., 2004), we adopted the individual I1 and I3 peak ISI from the measured SICF curve was used for both hemispheres in the protocol, as adjusting the individual I-wave peaks in iTMS has been reported to enhance iTMS concurrent MEP amplitude (Sewerin et al., 2011). To prevent iTMS-like facilitation accumulation in the measurement, paired-pulse stimuli were delivered every 10 s.

#### 2.3.3. TMS evaluation

TMS evaluation (in PA only, lasting approximately 10 min each) was performed at four timepoints (Figure 1, lower right panel). At the beginning of each TMS evaluation, 10 single-pulse stimuli with SI_0.5-1mV_ were delivered to the left hemisphere every 5 s. Subsequently, paired-pulse SICF-I1, SICF-I3 and LICF was measured in random order, with the ISI set as the individual I1/I3 peak for SICF-I1/I3 and 10 ms for LICF (Ilic et al., 2004; Delvendahl et al., 2014; Qasem et al., 2020). SICF intensity parameters were kept in line with the SICF curve measurement. The LICF intensity was set as CS = 70% rMT and TS = SI_0.5-1mV_ in a CS-TS order (Kujirai et al., 1993; Vucic et al., 2006; Wessel et al., 2019). Paired-pulse ICF was measured every 10 s, with a total of 10 trials each for SICF-I1, SICF-I3 and LICF. MEPs collected in each paradigm were averaged, from which the averaged MEP amplitude of MEP_sp._, SICF-I1, SICF-I3 and LICF was calculated, respectively. During the MEP measurement, subjects were instructed to maintain complete rest of their arms and hands.

After the MEP measurement, iSP of the left and right hemisphere (iSP_LH-RH_ and iSP_RH-LH_) was measured. Single-pulse TMS with SI_0.5-1mV_ was applied to the left and right hemisphere for 10 trials separately (applied every 5 s in each hemisphere), during maximum voluntary contraction (Giovannelli et al., 2009; Kuo et al., 2017) of the APB ipsilateral to the TMS pulse. During the ipsilateral contraction, participants were instructed to maintain complete rest of the contralateral hand, with bilateral EMG monitored by the experimenter. After removing the trials with no iSP presented, the EMG data was then averaged into one trial for automatic iSP detection (output as a duration value in milliseconds) in MATLAB using the same detection criteria as our previous publication (Tian et al., 2021). Additionally, to assess the IHI balance, IHI asymmetry ratio (IAR) was calculated according to the following formula (Tian et al., 2021):


$$\text{IAR}=\frac{{\text{iSP}}_{\text{LH-RH}}}{{\text{iSP}}_{\text{RH-LH}}}$$

In the formula, iSP_LH-RH_ represents iSP duration by left hemisphere TMS (inhibiting the right hemisphere), iSP_RH-LH_ represents iSP duration by right hemisphere TMS (inhibiting the left hemisphere). The higher the IAR value is, the stronger the IHI inhibiting the right hemisphere becomes, compared to the opposite direction. A value of 1 represents an IHI equivalency.

#### 2.3.4. iTMS intervention

In the iTMS intervention, repetitive paired-pulse TMS with identical pulse intensity (SI_0.5-1mV_) were delivered a 5-s inter-train interval (ITI) to the right hemisphere (Kidgell et al., 2016). For PA-iTMS, the TMS coil followed the setting of PA orientation, applying paired-pulse of SI_0.5-1mV_ (PA) at the individual I1 peak ISI. Similarly, AP coil positioning, SI_0.5-1mV_ (AP) intensity and I3 peak ISI was adopted in AP-iTMS. Due to the relatively high intensity of AP-iTMS, iTMS intensity was lowered to the highest comfort intensity according to the subject’s report, if the subject could not bear the SI_0.5-1mV_-AP intensity. Sham iTMS was performed using the PA-iTMS intensity and ISI, with the coil orientation altered as stated in 2.2.1 TMS equipment. ITMS was administered for 16 min in total (192 paired-pulses), with one TMS evaluation inserted in halfway of the intervention. To confirm complete rest of the muscles during iTMS and examine the ipsilateral online effects of iTMS as a premise for investigating the interhemispheric effects, bilateral EMG data (including MEP) in iTMS intervention was concurrently monitored and recorded. Concurrent MEP data during iTMS intervention was averaged into 32 blocks (6 paired-pulses each), and normalized as a percentage of averaged MEP of the first block. Due to the intensity lowering in AP-iTMS, subjects’ experiment data with AP-iTMS block 1 averaged MEP lower than 0.05 mV was excluded from further analysis.

### 2.4. Data processing and statistics

For the iTMS intervention, peak-to-peak amplitude of the MEP evoked by each paired-pulse was automatically calculated and extracted to a separate file using a customized LabChart 8.0 macro script, from which the block-averaged MEP amplitude was automatically calculated and then transferred to a general database for statistical analysis using a customized MATLAB script. For the TMS evaluation, the averaged MEP amplitude was automatically extracted and transferred to the general database for statistical analysis using a second LabChart macro script. Digitized raw EMG data of the averaged iSP trials was extracted to separate digital files using a third LabChart macro script for auto-calculation of iSP duration using customized MATLAB scripts. The iSP duration auto-calculated by MATLAB was double-checked by the experimenter to ensure data validity, and then transferred to the general database for statistical analysis.

To illustrate the direct effects of iTMS intervention on the right hemisphere, time-course change during iTMS was analyzed using two-way repeated measures analysis of variance (two-way rmANOVA), between interventions (AP-iTMS and PA-iTMS) and iTMS blocks (block 1–32). Furthermore, group data of 32-blocks-averaged MEP amplitude in the two real iTMS interventions was also analyzed by linear regression (Thickbroom et al., 2006; Silbert et al., 2011).

For TMS evaluation, MEP_sp_ amplitude of the left hemisphere measured at each timepoint was included in the analysis. ICF was normalized to the MEP_sp_ amplitude at each timepoint. Baseline intra-subject difference of the three interventions was analyzed using one-way rmANOVA, with INTERVENTION (PA-iTMS, AP-iTMS, sham-iTMS) set as within-subject factor. To test the interhemispheric iTMS effects on the facilitatory circuits, MEP_sp_ and ICF outcomes were entered as dependent variables and analyzed using two-way multivariate analysis of variance (two-way MANOVA), with TIME (baseline, during, post0’, post10’) and INTERVENTION (PA-iTMS, AP-iTMS, sham-iTMS) set as within-subject factors. ISP duration was automatically calculated from the raw EMG signal using customized MATLAB script and expressed as a duration in milliseconds, based on which IAR was then calculated. In summary, MEP_sp_ amplitude, ICF ratio (SICF-I1, SICF-I3, LICF); iSP duration (iSP_LH-RH_, iSP_RH-LH_) and IAR collected from TMS evaluation were included in the statistical analysis.

Statistical analysis was conducted using the Statistical Package for the Social Sciences (SPSS) version 20.0 for Windows (IBM Corp., Armonk, NY, United States). Statistical significance was denoted at *p* < 0.05. Following statistically significant results, *post hoc* test using Bonferroni’s correction was performed. Similarly, to unveil the IHI modulation, iSP_LH-RH_, iSP_RH-LH_ and IAR were also analyzed by two-way MANOVA, with the same within-subject factor set as aforementioned. Additionally, to address the specific time-course change in each intervention, a follow-up one-way rmANOVA was performed with the three iTMS interventions analyzed independently. If significant baseline difference was detected, one-way analysis of covariance (ANCOVA) was used in substitution for rmANOVA to analyze the time-course change, with the baseline value of the parameter adjusted as the co-variate. Figures were generated using customized MATLAB 2021b scripts.

## 3. Results

### 3.1. Baseline parameters

**Figure 2:**
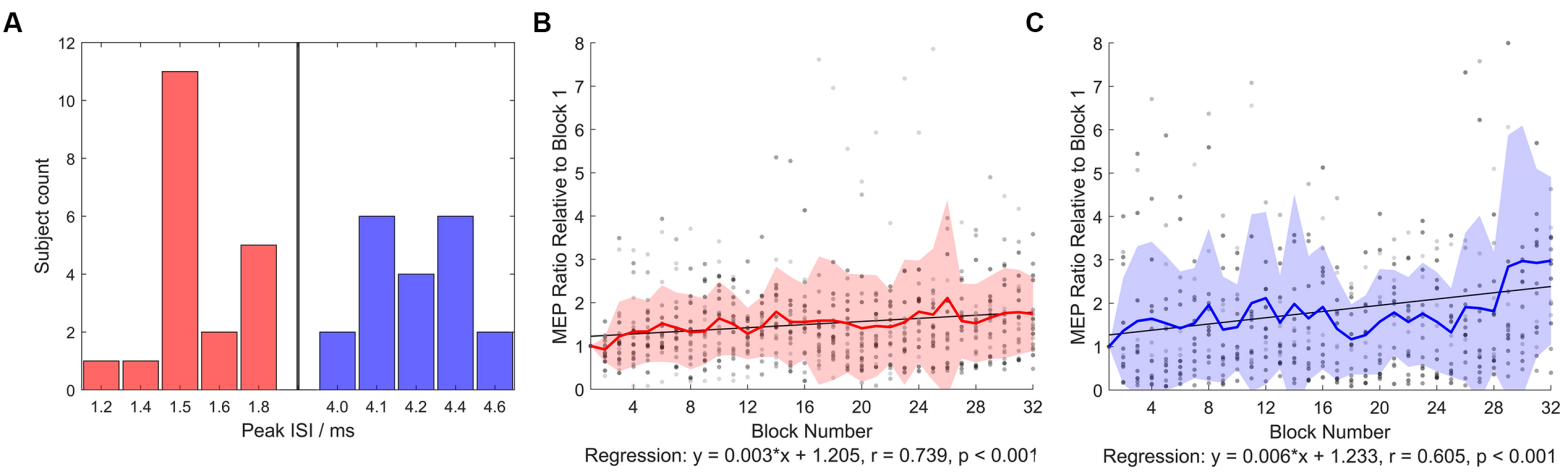
SICF peak ISI histogram and normalized iTMS concurrent MEP. **(A)** Number of subjects showing peak ISIs in the SICF curve measurement (from which ISI in iTMS and SICF evaluation was used) was plotted. Red bars denote early I-wave timings, blue bars denote late I-wave timings. **(B)** Normalized PA-iTMS concurrent MEP (*N* = 20). The iTMS intervention was divided into 32 blocks (6 paired pulses averaged per block), and the evoked MEP amplitude was normalized to block 1 averaged MEP. **(C)** Normalized AP-iTMS concurrent MEP (*N* = 15). Both the PA- and AP-iTMS potentiated MEP in the right hemisphere steadily, yet with considerable individual variability. Greyscale dots denote individual data. Bold colored line denotes group mean. Filled area denotes mean ± standard deviation (SD) range.

The experiment protocol was well-tolerated by all subjects (*N* = 20). No side-effect was reported throughout the experiment (from the first visit to 1 week after the last visit). TMS intensities in each intervention was summarized in Table 1. In AP-iTMS, nine out of 20 subjects were not able to tolerate the intensity of AP-SI_0.5-1mV_, therefore the AP-SI_0.5-1mV_ was lowered to the highest comfort intensity (Table 1). Among the nine subjects with lowered stimulation intensity in AP-iTMS, five subjects’ AP session data was excluded from all analysis, as their AP-iTMS block 1 averaged MEP amplitude was lower than 0.05 mV. Histogram of the individual SICF peak ISIs (which were used for iTMS and TMS evaluation) was shown in Figure 2A.

**Table 1:** Mean (SD) of the TMS parameters (stimulation intensity) in each intervention.

TMS parameters (% MSO)	PA-iTMS (*N* = 20)	AP-iTMS (*N* = 15)*	Sham-iTMS (*N* = 20)
rMT-LH	50.6 (8.4)	51.7 (9.0)	50.5 (7.9)
rMT-RH-PA	50.3 (8.2)	51.7 (7.6)	53.1 (8.8)
rMT-RH-AP	N/A	67.0 (5.2)	N/A
SI_0.5-1mV_-LH	63.5 (8.8)	65.2 (8.7)	64.4 (8.2)
SI_0.5-1mV_-RH-PA	66.4 (8.4)	65.1 (7.8)	67.2 (8.1)
SI_0.5-1mV_-RH-AP	N/A	74.4 (5.9)	N/A

rMT, resting motor threshold; SI_0.5-1mV_, TMS stimulus intensity that evoked 0.5-1 mV MEP; AP/PA, anteroposterior/posteroanterior (induced current direction); LH, left hemisphere; RH, right hemisphere; % MSO, percentage of maximum stimulator output; N/A, not applicable. *The AP SI_0.5-1mV_ in nine out of 20 subjects were lowered as the subjects could not bear the expected SI_0.5-1mV_ intensity, among which the data of five subjects with AP-iTMS block 1 average MEP amplitude <0.05 mV was removed from all analysis. Values in this column are lowered values of 15 subjects whose data was included in the statistical analysis.

No baseline intrasubject difference was found in MEP_sp_ amplitude, SICF-I1, iSP_LH-RH_, iSP_RH-LH_ and IAR (rmANOVA, *p* > 0.05). However, significant intrasubject difference of baseline SICF-I3 and LICF ratio was detected between PA-iTMS and sham-iTMS (*p* = 0.018 for SICF-I3; *p* = 0.034 for LICF). The outcome of the two parameters in PA-iTMS was analyzed by ANCOVA with baseline value adjusted.

### 3.2. TMS concurrent MEP

At the first block of iTMS, averaged paired-pulse MEP amplitude was 0.7 ± 1.3 mV in PA-iTMS and 0.2 ± 0.1 mV in AP-iTMS (as the stimulation intensity was lowered). At the final block, averaged paired-pulse MEP amplitude was 1.1 ± 2.1 mV in PA-iTMS and 0.5 ± 0.4 mV in AP-iTMS. In sham-iTMS, no MEP was elicited throughout the intervention (all EMG amplitude <0.05 mV).

Two-way rmANOVA revealed significant main effects of BLOCK (*F*_31,1,037_ = 2.664, *p* < 0.001) and INTERVENTION (*F*_1,1,037_ = 29.732, *p* < 0.001). No significant BLOCK × INTERVENTION interaction was detected (*p* = 0.125). Regression analysis of iTMS concurrent MEP revealed significant time-course change during PA- and AP-iTMS (Figures 2B,C). In PA-iTMS, the averaged MEP ratio showed a steady increase (*N* = 20, *r* = 0.739, *F*_1,30_ = 36.143, *p* < 0.001), yielding a 173.8 ± 85.6% increase at the end. In AP-iTMS, a significant increase in the MEP amplitude was also observed (*N* = 15, *r* = 0.605, *F*_1,30_ = 17.329, *p* < 0.001) with a 297.9 ± 192.9% increase.

### 3.3. Interhemispheric effects of iTMS: overall

Two-way MANOVA revealed significant effects of TIME (Wilks’ *λ* = 0.781, *F*_10,408_ = 5.364, *p* < 0.001) and INTERVENTION (Wilks’ *λ* = 0.866, *F*_15,563.556_ = 2.014, *p* = 0.013) on the overall MEP data (MEP_sp._, SICF-I1, SICF-I3 and LICF), without a significant INTERVENTION × TIME interaction (Wilks’ *λ* = 0.910, *F*_30,818_ = 0.651, *p* = 0.926). For IHI data (iSP_LH-RH_, iSP_RH-LH_ and IAR), a significant effect of INTERVENTION (Wilks’ *λ* = 0.843, *F*_6,412_ = 6.129, *p* < 0.001) was also found. No significant effect of TIME was revealed in IHI parameters (Wilks’ *λ* = 0.977, *F*_9,501.5_ = 0.540, *p* = 0.846). Particularly, albeit the overall INTERVENTION × TIME interaction (Wilks’ *λ* = 0.875, *F*_18,583.141_ = 1.572, *p* = 0.062) failed to reach statistical significance, an INTERVENTION × TIME interaction on IAR inter-subject difference was significant (*F*_6,208_ = 4.270, *p* < 0.001). In sham-iTMS, no significant time-course change in any of the analyzed parameters were detected (all *p* > 0.05, Table 2).

**Table 2:** Mean (SD) of the MEP_sp_ and ICF from the TMS evaluation of sham-iTMS.

Parameter	Baseline	During	Post0’	Post10’	*F*_3,57_	*p*
MEP_sp_/mV	0.905 (0.46)	0.846 (0.39)	0.892 (0.44)	0.913 (0.45)	0.760	0.521
SICF-I1	1.275 (0.45)	1.276 (0.52)	1.233 (0.43)	1.305 (0.45)	0.214	0.886
SICF-I3	1.127 (0.28)	1.116 (0.36)	1.147 (0.35)	1.247 (0.38)	1.225	0.309
LICF	1.229 (0.34)	1.182 (0.48)	1.168 (0.38)	1.285 (0.53)	0.507	0.679
iSP_LH-RH_/ms	36.05 (9.85)	35.95 (9.73)	35.30 (9.80)	36.05 (10.66)	0.084	0.969
iSP_RH-LH_/ms	31.30 (7.39)	32.65 (9.91)	33.05 (10.60)	32.25 (9.71)	0.553	0.648
IAR	1.176 (0.30)	1.163 (0.43)	1.117 (0.27)	1.169 (0.32)	0.198	0.897

MEP, motor evoked potential; SICF-I1/SICF-I3, short-interval intracortical facilitation at I1/I3 peak ISI; LICF, long-interval intracortical facilitation; iSP, ipsilateral silent period; IAR, interhemispheric inhibition asymmetry ratio; RH/LH, right/left hemisphere; sp., single-pulse.

### 3.4. Interhemispheric effects of iTMS: facilitatory circuits

**Figure 3:**
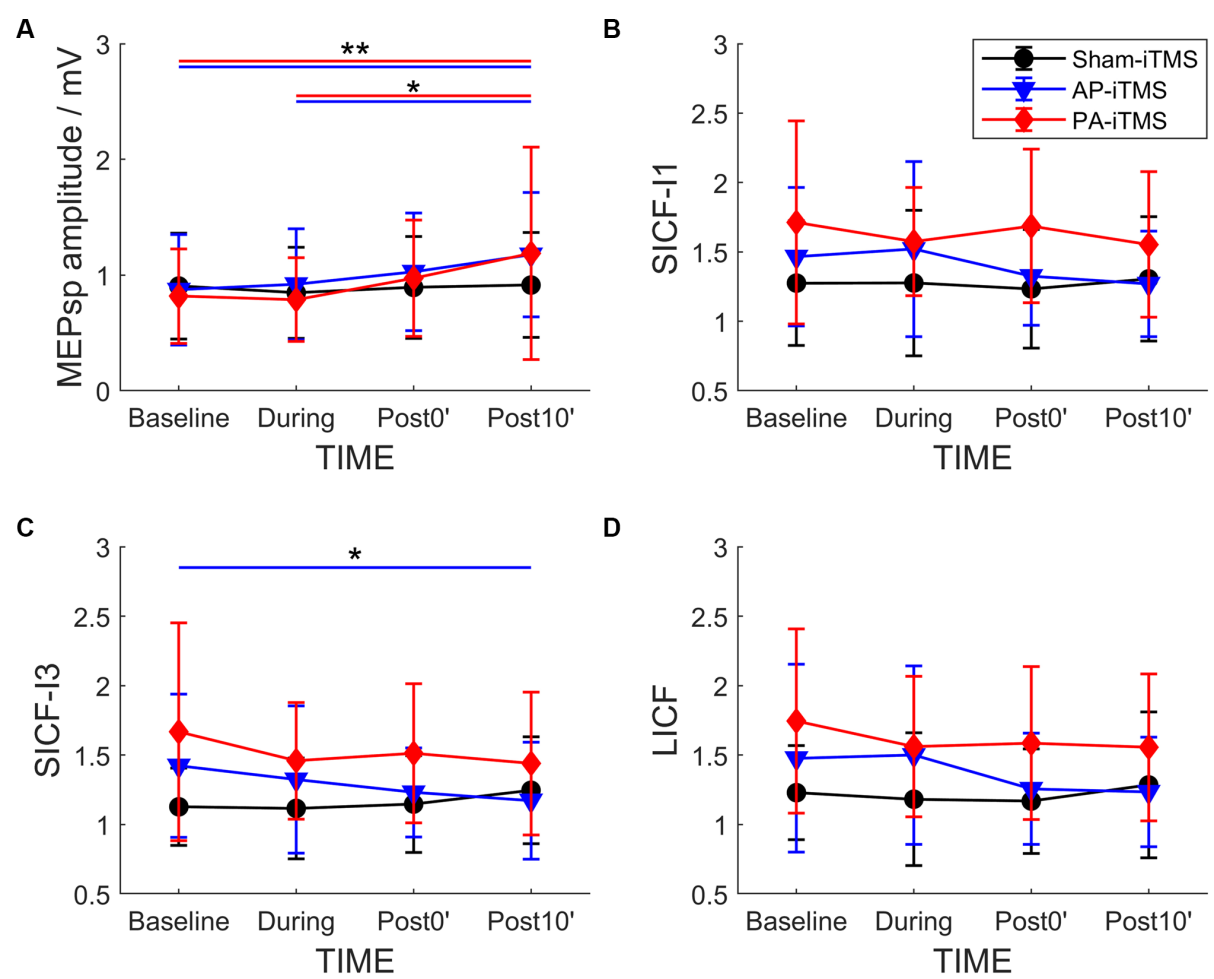
Time-course change of MEP_sp_ amplitude **(A)**, SICF-I1 **(B)**, SICF-I3 **(C)** and LICF **(D)** ratio in the three interventions. Shapes and error bars denote mean ± SD. *p < 0.05; **p < 0.01.

The independent rmANOVA of PA-iTMS revealed a significant effect of TIME on MEP_sp_ amplitude (*F*_3,57_ = 4.927, *p* = 0.004), but not in SICF-I1 (*F*_3,57_ = 0.553, *p* = 0.648) (Figure 3). *Post-hoc* test revealed that: (1) MEP_sp_ amplitude at post10’ was significantly higher than that at baseline (*p* = 0.015) and during (*p* = 0.007) timepoints. For SICF-I3 and LICF with a significantly higher baseline than AP- and sham-iTMS, baseline-adjusted ANCOVA revealed no effect of TIME on the two parameters (all *p* > 0.05) in PA-iTMS.

In AP-iTMS, rmANOVA revealed a significant effect of TIME on MEP_sp_ amplitude (*F*_3,42_ = 7.857, *p* < 0.001) and SICF-I3 (*F*_3,42_ = 3.225, *p* = 0.032) (Figure 3). *Post hoc* test revealed that MEP_sp_ amplitude at Post10 timepoint was largNer than that at Baseline (*p* < 0.001) and During (*p* = 0.004) timepoints. SICF-I3 at Post10 timepoint was significantly lower compared to Baseline timepoint (*p* = 0.021).

### 3.5. Interhemispheric effects of iTMS: interhemispheric inhibitory circuits

**Figure 4:**
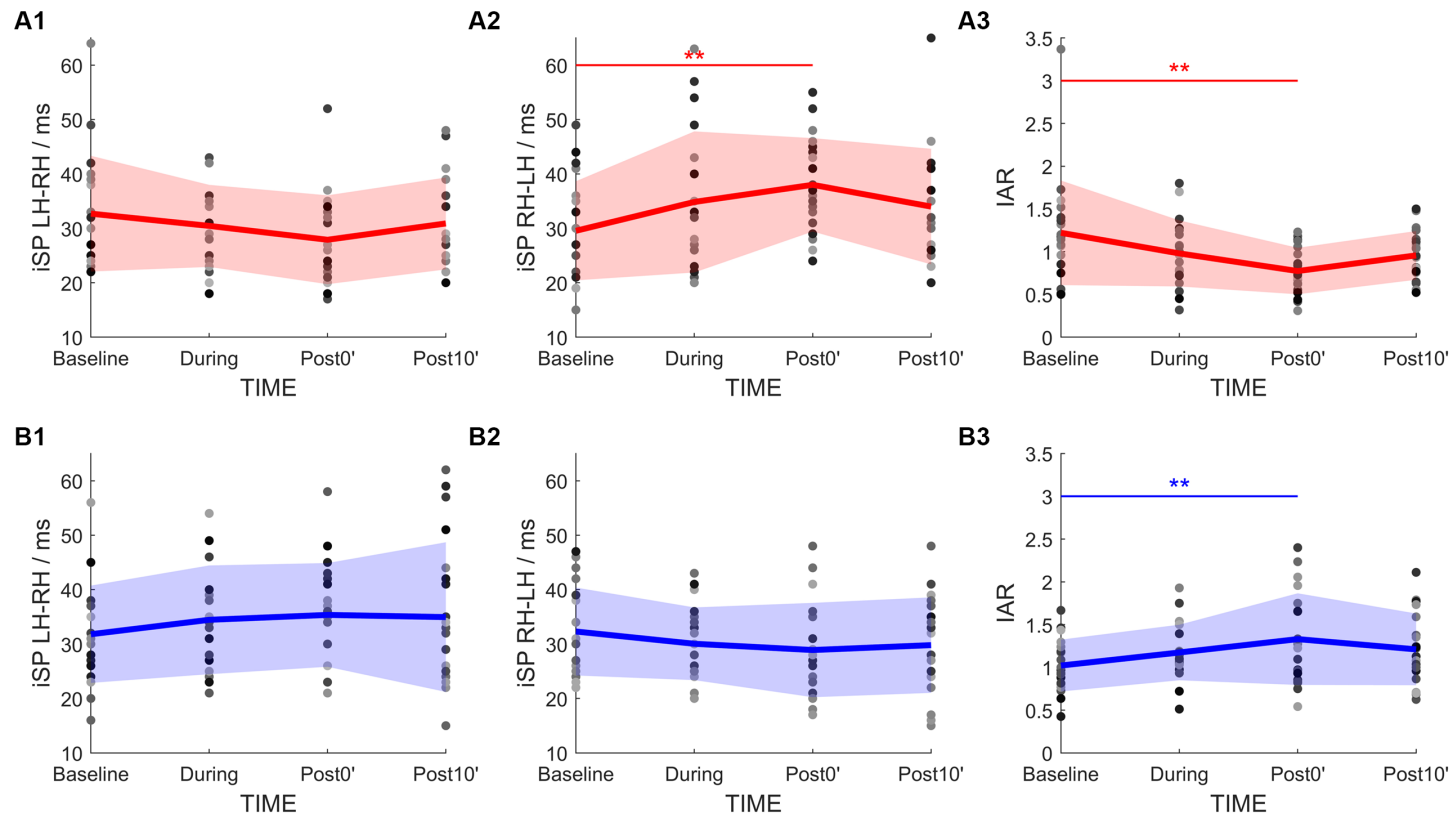
Time-course change of the IHI parameters in PA-iTMS (red, **A1–A3**) and AP-iTMS (blue, **B1–B3**). Left column, iSP duration by left hemisphere TMS (iSP_LH-RH_). Middle column, iSP duration by right hemisphere TMS (iSP_RH-LH_). Right column, IHI Asymmetry Ratio (IAR) calculated from bilateral iSP. Greyscale dots denote individual data. Bold lines denote group mean. Colored areas denote mean ± SD range. *p < 0.05; **p < 0.01.

rmANOVA of PA-iTMS demonstrated a significant effect of TIME on iSP_RH-LH_ (*F*_3,57_ = 4.271, *p* = 0.009) and IAR (*F*_3,57_ = 5.567, *p* = 0.002, Figures 4A2,A3), while iSP_LH-RH_ showed no statistical significant effect of TIME (*F*_3,57_ = 1.764, *p* = 0.164, Figure 4A1). *Post-hoc* test revealed that: (1) iSP_RH-LH_ at post0’ was significantly longer than that at baseline (*p* = 0.005); and (2) IAR at post0’ was significantly lower than that at baseline (*p* = 0.001).

For AP-iTMS, a main effect of TIME was found in IAR only (*F*_3,42_ = 5.454, *p* = 0.003, Figure 4B3), being the IAR at post0’ significantly higher than that at baseline (*p* = 0.002) in the *post-hoc* test. No effect of TIME was found in bilateral iSP (iSP_LH-RH_: *F*_3,42_ = 1.615, *p* = 0.200, Figure 4B1; iSP_RH-LH_: *F*_3,42_ = 2.202, *p* = 0.102, Figure 4B2).

## 4. Discussion

**Figure 5:**
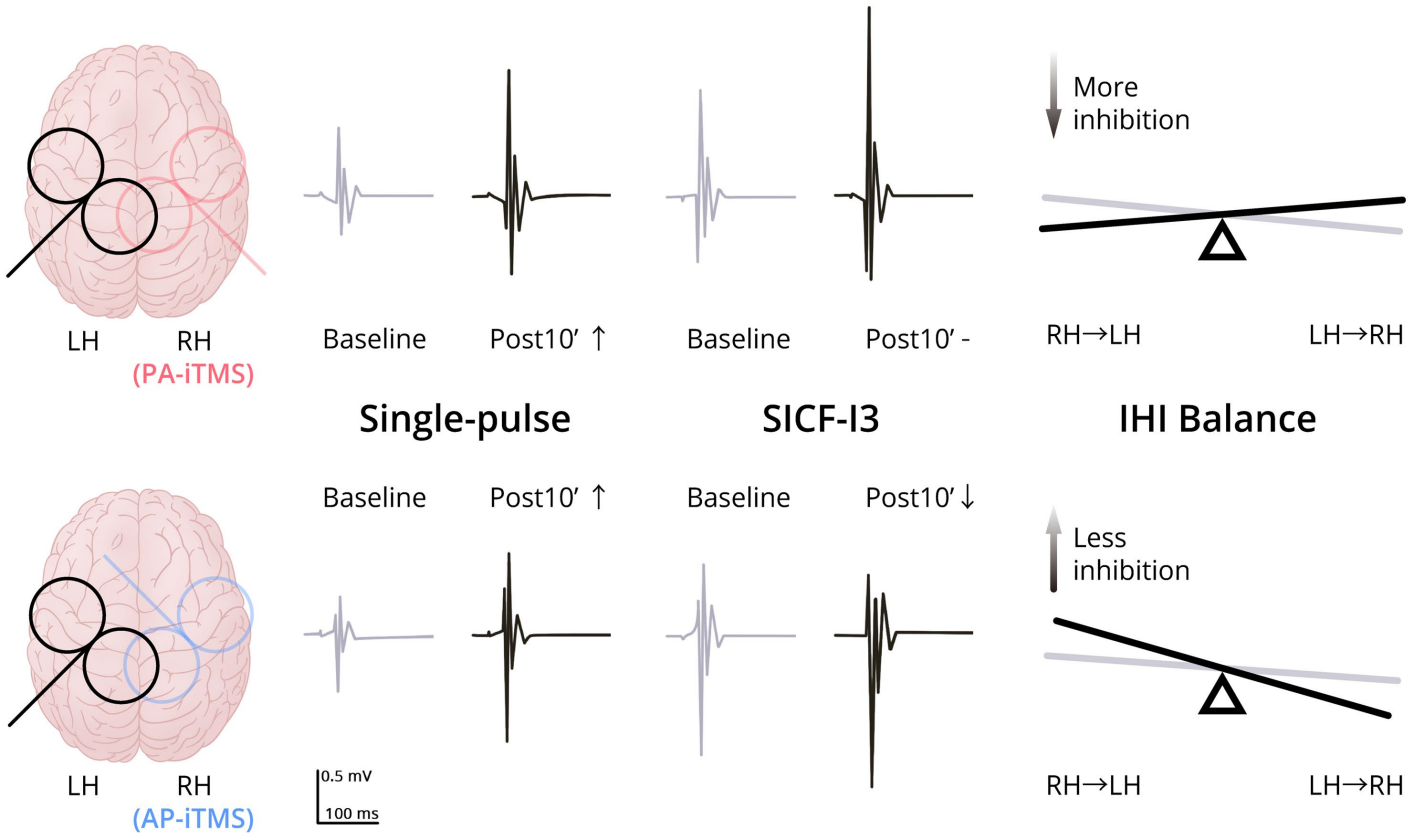
Schematic summary of the present results. Upper row, results of PA-iTMS; Lower row, results of AP-iTMS. Left column, coil placement (overlapped) of TMS evaluation (black) and iTMS intervention (red for PA-iTMS, blue for AP-iTMS). Middle column, typical MEP change of single-pulse TMS and paired-pulse SICF-I3 in the non-stimulated hemisphere at Baseline (grey) and Post10’ (black). Both the PA-iTMS and AP-iTMS potentiated single-pulse MEP (↑) in the non-stimulated hemisphere, yet AP-iTMS decreased the facilitation of SICF-I3 (↓), differing from PA-iTMS which did not (-). Right column, change of the IHI balance in the two interventions from Baseline (grey) to Post0’ (black). PA-iTMS tilted the IHI balance towards more inhibition targeting the non-stimulated left hemisphere by increasing iSP in the right hemisphere, whereas AP-iTMS shifted the balance to the opposite direction. Abbreviations: LH = left hemisphere; RH = right hemisphere; PA/AP = posterolateral-anteromedial/anteromedial-posterolateral (TMS coil orientation); SICF = short-interval intracortical facilitation; IHI = interhemispheric inhibition.

In the present study, we explored the effects on interhemispheric interaction by iTMS in different induced current directions. We found that: (1) both PA-iTMS and AP-iTMS potentiated the excitability of the non-stimulated motor cortex as assessed by single-pulse TMS; and (2) the change of IHI and ICF in the non-stimulated hemisphere differed between PA- and AP-iTMS. Figure 5 summarizes the primary results of the present study.

### 4.1. iTMS concurrent effect

iTMS has been considered to induce LTP-like effects in the cortical I-wave circuits as the ISI of iTMS corresponds with the timing of I-waves in epidural recordings (Thickbroom et al., 2006). The facilitation of iTMS originates at the cortical level, as iTMS did not facilitate the brainstem MEPs (Hamada et al., 2007) or the cervico-medullary junction composite MEPs (Di Lazzaro et al., 2007). In the present study, the excitability of the right hemisphere was upregulated by both PA- and AP-iTMS and reflected in the steady increase of the concurrent MEPs, corresponding to previous reports from PA-iTMS studies (Murray et al., 2011; Sewerin et al., 2011; Teo et al., 2012; Kidgell et al., 2016). Regarding AP-iTMS, we observed a steady MEP potentiation similar to PA-iTMS. However, although the degree of increase seems to be higher in AP-iTMS than PA-iTMS, this difference may be a result of baseline MEP difference and should be interpreted with caution, as not all baseline MEP was adjusted to 0.5–1 mV. Therefore, we believe that it is imprudent to compare the efficiency of AP- and PA-iTMS in the present study, given the obvious difference in baseline MEP amplitudes as well as the smaller sample size in AP-iTMS due to the exclusion of five subjects’ data.

### 4.2. iTMS interhemispheric effect: similarities and differences

In the present study, we identified similar interhemispheric facilitatory effect on MEP_sp_ by PA- and AP-iTMS, together with different modulation on ICF and IHI. Our results are consistent with the reports showing MEP increase in the non-stimulated hemisphere after 20-Hz rTMS, 5-ms QPS and 25-ms paired associative stimulation (PAS_25_) (Di Lazzaro et al., 2011; Tsutsumi et al., 2013; Tian and Izumi, 2022a). Meanwhile, positron emission tomography (PET), functional magnetic resonance imaging (fMRI) and functional near-infrared spectroscopy (fNIRS) studies have also reported bilateral excitability potentiation by excitatory rTMS (Siebner et al., 2000; Watanabe et al., 2014; Li et al., 2019; Honda et al., 2021). These observations indicate the existence of an interhemispheric facilitation mechanism between the hemispheres, in addition to the well-investigated interhemispheric inhibition mechanism (Di Lazzaro et al., 2008). While the interhemispheric interaction mechanism is complicated and remains largely unknown at present, the existence of both inhibitory and excitatory circuits seems evident, and it is possible that the two mechanisms work in separate regimes. Indeed, in the present study, although IHI changed immediately after the intervention, the effect on MEP emerged 10 min after PA- and AP-iTMS. The change is congruent with previous studies showing a delayed plasticity emergence in iTMS (Thickbroom et al., 2006) and other NIBS protocols (Batsikadze et al., 2013; Davila-Pérez et al., 2019; Nakazono et al., 2021). The timing of IHI (including IAR) change prior to MEP potentiation may suggest a faster response of the IHI circuits to contralateral iTMS than the excitatory circuits, which in turn supports the “excitatory transcallosal projection onto local interneurons (INs)” theory of the IHI circuits.

Conversely, effect difference on ICF and IHI was identified as: (1) AP-iTMS suppressed SICF-I3 in the non-stimulated hemisphere, and (2) PA-iTMS tilted the IHI balance toward inhibiting the non-stimulated hemisphere, while AP-iTMS tilted the balance to the opposite direction. Although the evidence regarding SICF modulation by rTMS is highly inconsistent (Fitzgerald et al., 2006), we found it intriguing that SICF-I3 was suppressed by AP-iTMS. As the ISI of SICF-I3 is approximately 4.5 ms, which is approximately ~3 synapse conduction time (Ziemann, 2020; Tian and Izumi, 2022b; Tian and Izumi, 2023), the results suggest that the facilitation from paired-pulse SICF-I3 might be generated from longer neuron chains with multiple synapses, with the neurons on the chain bearing more inhibition from local INs (Peurala et al., 2008). This effect can become greater if the I3 circuitry is specifically targeted by AP-iTMS. For the modulation of IHI, it has long been evidenced that excitatory rTMS increases the inhibition targeting the non-stimulated hemisphere (Cincotta et al., 2006; Tsutsumi et al., 2013; Hui et al., 2020; Bai et al., 2021). We also found a significant increase of the iSP from the stimulated to the non-stimulated hemisphere after PA-iTMS. Interestingly however, AP-iTMS directed IHI toward the opposite of PA-iTMS by tipping the IHI balance toward a weaker inhibition from the stimulated hemisphere, without directly altering the iSP outcome. Further discussions on the neural substrates underlying AP-iTMS are included in the next section.

### 4.3. iTMS neurocytological mechanism model

**Figure 6:**
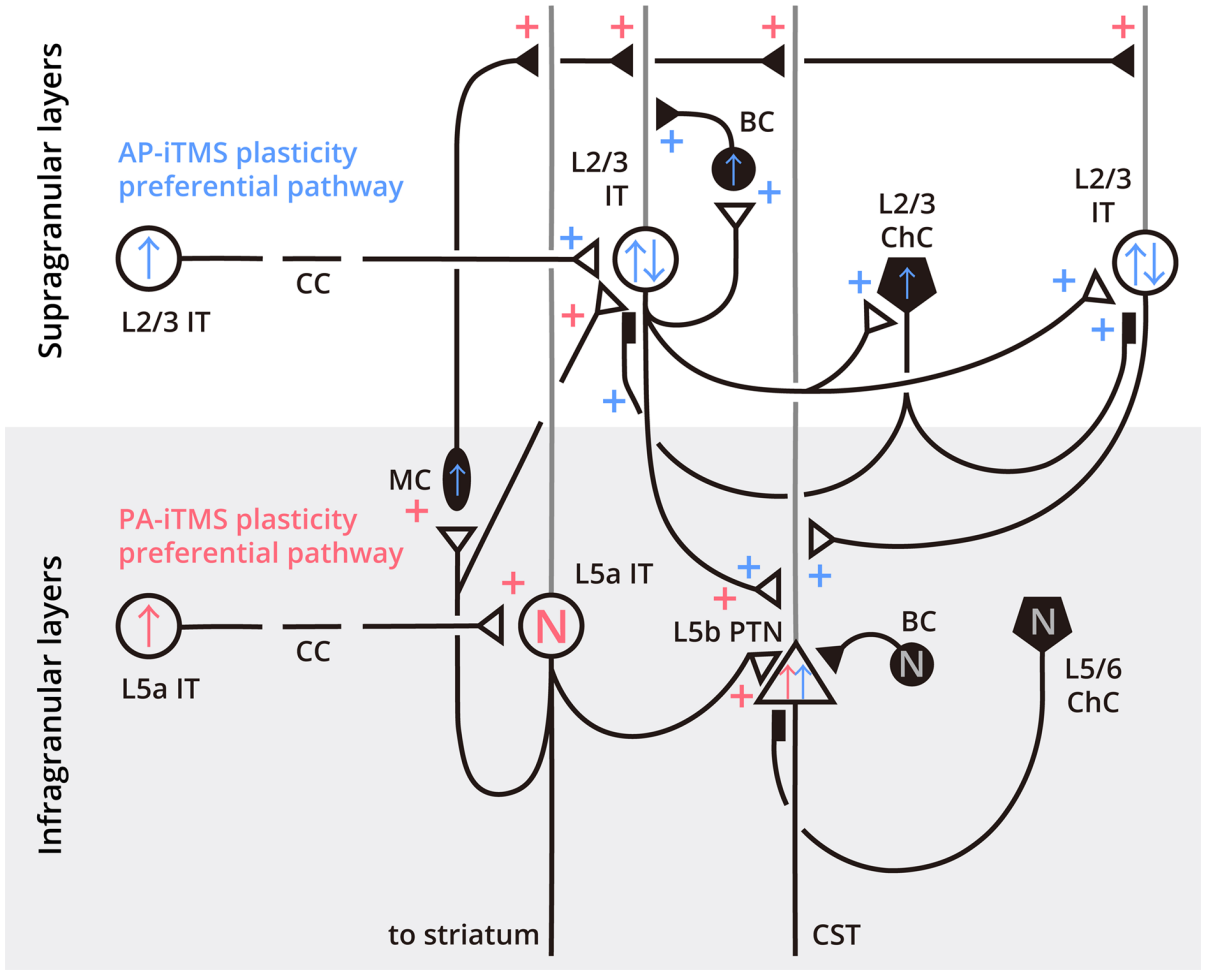
Neurocytological model of the preferential pathways of PA- and AP-iTMS neuroplasticity in the primary motor cortex. Cortical layers are divided into supragranular (white background) and infragranular (light-gray background) layers, with the layer (L) of the specific neuron labeled next to the cell soma. Open circles indicate intratelencephalic projecting neurons (IT), open triangles indicate pyramidal tract neurons (PTN). Filled circles, ovals and pentagons denote inhibitory interneurons as basket cells (BC), Martinotti cells (MC) and chandelier cells (ChC), respectively. Black lines with small triangles denote axons and synapses, gray lines denote apical dendrites of the pyramidal neurons. Filled rectangles indicate the special synaptic connection between ChC and pyramidal neurons at the axonal initial segment. The plastic effects of AP-iTMS are colored in blue, while the PA-iTMS-related effects are colored in red. Plus symbols next to the synapses indicate LTP, arrows inside the cell soma indicate the overall neuronal excitability change, as ↑ (increase), ↓ (decrease) or N (not changed). L2/3 ITs labeled with blue “↑↓” symbols denote that in AP-iTMS, although the excitability of L2/3 ITs were initially potentiated by the transcallosal LTP, overall excitability of the ITs was counteracted by feedback inhibition, resulting in little change in SICF-I1. In longer IT chains involving more inhibitory INs, the increase of the IN excitability can cause more inhibition and interfere with the facilitation of SICF-I3, leading to its decrease in the experimental results. CC, corpus callosum; CST, corticospinal tract.

The present results indicate that the neural circuits involved in AP-iTMS differ from that in PA-iTMS. Evidence has suggested that reversing TMS-induced current direction from PA to AP may cause opposite activation order of the anterior bank (mainly dorsal premotor cortex) and the posterior bank (mainly M1) of the precentral gyrus (Di Lazzaro et al., 1998; Siebner et al., 2022). This could result in different recruitment of pyramidal neurons and INs in the supragranular (integrating inputs from other areas) and infragranular layers (constituting the motor output to subcortical regions) in the motor cortex (Figure 6; Szentagothai and Arbib, 1974; Reiner et al., 2003; Inan and Anderson, 2014; Callaway et al., 2021). In the present hypothetical model, the six-layered cortical cytoarchitecture is divided into supragranular layers (layers I-III) and infragranular layers (layers IV-VI). Main neuron subtypes included in the model are excitatory pyramidal neurons with projections targeting bilateral cortex and striatum (i.e., intratelencephalic projecting neurons, ITs), excitatory PTNs constituting the corticospinal tract, and inhibitory INs targeting local excitatory ITs and PTNs. Interconnected IT neurons in the supragranular layers (L2/3 ITs) targeting PTNs at layer Vb (L5b PTN) are considered forming neuron chains responsible for SICF (L2/3 IT-L5b PTN chain for SICF-I1, and L2/3 IT-(multiple) L2/3 IT-L5b PTN chain for SICF-I3, Figure 6), with an approximately 1.5-ms delay for each synaptic connection (Sjöström and Häusser, 2006). For LICF, empirical evidence regarding its mechanisms is still lacking. However, as proposed in our previous model LICF might be related to the timing-dependent inhibition-excitation shift of chandelier cells (ChCs, a subtype of cortical INs) on the postsynaptic neurons (Woodruff et al., 2011; Tian and Izumi, 2022b; Tian and Izumi, 2023). For INs, based on quantitative proportion (Rudy et al., 2011; Bakken et al., 2021), two main IN subtypes related to intracortical inhibition are included in the model, being the basket cells (BCs) and ChCs. In particular, BCs mainly target the cell soma of the postsynaptic neurons (Micheva et al., 2021), while ChCs form special synapses with the postsynaptic neurons at the axonal initial segment (Inan and Anderson, 2014). Moreover, the inhibitory Martinotti cells mediating lateral inhibition is also included as they have been considered responsible for IHI (Obermayer et al., 2018). Based on the present results, we propose that AP-iTMS preferentially induces LTP in cortical L2/3 IT neurons while PA-iTMS selectively affects ITs in deeper layers (e.g., L5a ITs), through homologous neuron connections. Accordingly, we infer the mechanisms of iTMS based on the neuron connectivity reported by animal studies and our previous model (Szentagothai and Arbib, 1974; Reiner et al., 2003; Silberberg and Markram, 2007; Inan and Anderson, 2014; Sohur et al., 2014; Lin et al., 2018; Tasic et al., 2018; Zolnik et al., 2020; Callaway et al., 2021; Tian and Izumi, 2022b), as shown in Figure 6.

In AP-iTMS, we observed an increase of corticospinal excitability, a decrease in the facilitation of SICF-I3, and no modulation of LICF in the non-stimulated hemisphere. Given the excitatory characteristics of callosal projections (Conti and Manzoni, 1994), it can be presumed that LTP is relayed to the non-stimulated hemisphere transcallosally in both protocols. As AP-iTMS altered SICF, LTP can be considered relayed from the stimulated hemisphere mainly to L2/3 ITs responsible for SICF (the IT-PTN or IT-IT-PTN circuit), causing an initial increased response of the ITs. The potentiated ITs further relays LTP through its axons onto other L2/3 ITs, L5b PTNs and L2/3 INs such as BCs and ChCs (responsible for short-interval intracortical inhibition, SICI). The initial potentiation of L2/3 ITs is then counteracted due to feedback inhibition by BCs and ChCs, resulting in little change in SICF-I1. However, in longer IT chains involving more inhibitory INs, the increase of IN excitability can cause more inhibition and interfere with the facilitation of SICF-I3. For PTNs in the infragranular layers, as the excitability of deep layer INs is not altered, the potentiation targeting L5 PTNs remains and results in the MEP_sp_ amplitude increase. Although the excitability of L2/3 INs including ChCs is upregulated in the model, the lack of LICF change might result from the difference in the ChC effect timing, which is related to the difference of glutamate and GABA receptors activity. That is, a change of SICI mediated by ChCs may not be reflected when assessed with the LICF paradigm, as the two phenomena differ in neurotransmitters. For IHI, in AP-iTMS, we only observed subtle change in the IHI balance, without significant modulation of bilateral iSP. Therefore, it is possible that AP-iTMS LTP does not directly affect the activity of Martinotti cells, thus leading to no direct change of IHI. The change of IHI balance in AP-iTMS may be due to a subtle IHI reduction from the stimulated to the non-stimulated hemisphere, since the balance ratio IAR is more sensitive to detect subtle change of the IHI balance. This subtle reduction of IHI targeting the non-stimulated hemisphere may be due to a relative decrease of transcallosal LTP relay between deep-layer ITs, for the excitability of the supragranular ITs and its transcallosal projections is mainly potentiated by AP-iTMS.

Conversely, in PA-iTMS, while we observed an increase in corticospinal excitability similar to AP-iTMS, no alteration in SICF was observed. Simultaneously, IHI inhibiting the non-stimulated hemisphere increased significantly. Accordingly, we infer that PA-iTMS mainly causes LTP in the infragranular callosal-projecting neurons, most typically L5a ITs, which are reported to be directly engaged in lateral inhibition by Martinotti cells (Jiang et al., 2015). Apart from modulating the activities of Martinotti cells, the L5a ITs also relay LTP to L5b PTNs directly via local IT-PTN connections (Kim et al., 2015; Baker et al., 2018; Im et al., 2023), and to L2/3 ITs through translaminar projections (Silberberg and Markram, 2007). While the LTP relayed through L5a IT-L2/3 IT connections follows the same pathway as AP-iTMS, the LTP relayed through the local connections between L5a IT and L5b PTN can bypass the L2/3 IT-mediated SICF circuitry, and results in direct potentiation of the L5b PTNs. As a result, these neuronal pathways account for the increase of corticospinal excitability in the non-stimulated hemisphere without affecting SICF.

### 4.4. Significance and limitations

Even though the effect of inhibitory rTMS has been evidenced to facilitate poststroke rehabilitation by rebalancing the IHI (Pal et al., 2005; Boddington and Reynolds, 2017), recent insights on facilitating the non-lesioned hemisphere to promote function compensation is gaining interest and attention (Wang et al., 2020). Apart from suppressing the unaffected hemisphere according to the interhemispheric competition model, a bilateral cortical activation might be more effective for stroke recovery and functional compensation in patients with severe functional impairment and little residual networks substitutes (Di Pino et al., 2014; Lin et al., 2020). We believe that our findings on the interhemispheric facilitatory effects of iTMS can inspire both basic studies exploring the neurobiological circuit wiring of human cerebral cortex and clinical practice in neurorehabilitation.

Nevertheless, our study is limited in a few different ways. A first notable limitation is the insufficiency of sample size and stimulation intensity in AP-iTMS stimulation. In the present study, five out of 20 subjects’ AP-iTMS results were excluded from the analysis, leaving only 15 subjects included in the AP-iTMS results analyses. Therefore, our findings may suffer from bias caused by this small sample size, which demand examination in future studies. Additionally, given the nonlinear nature of the TMS input–output curve (Rossini et al., 2015), normalization based on smaller MEPs (evoked by lower TMS intensities) may bear the risk of overestimating the actual effect. Consequently, comparison of the online effects of PA- and AP-iTMS demands further investigation. Secondly, the voluntary muscle contraction in iSP measurement during the TMS evaluation may cause contamination of the iTMS intervention effects. As voluntary contraction represents a form of cortical activation, it can therefore affect inhibitory rTMS protocols such as continuous TBS (cTBS), either abolishing the inhibitory aftereffects (Huang et al., 2008; Goldsworthy et al., 2014, 2015) or reversing the effects into facilitation (Gentner et al., 2008; Iezzi et al., 2008). In excitatory protocols, the observations differ between protocols. While Huang et al. (2008) reported further enhancement of iTBS facilitatory effect when contraction was made immediately after iTBS, Kadowaki et al. (2016) reported from their QPS study that contraction immediately after the intervention abolished the effects of both excitatory QPS_5_ and inhibitory QPS_50_. Although the cause of this inconsistency has not been clarified yet, it is however clear that voluntary movement has an impact on the plasticity effects induced by rTMS, which should be noted in the present study as a limitation. Thirdly, in the SICF curve measurement, only five MEPs were acquired in each ISI. As at least 30 trials are necessary to provide a reliable estimate of ICF (Biabani et al., 2018), it is highly possible that we did not perform an accurate measurement of the SICF peaks in our study, and in turn did not execute the “adjusting the individual I-wave peaks” properly. That is, we are currently not able to make any inference regarding whether the iTMS effects were enhanced, for the lack of reliability on the SICF peaks may affect the iTMS concurrent effects in both PA- and AP-iTMS. Consequently, we observed considerable inter-individual variation in the present results. Therefore, our discovery may subject to problems with type I error. Further studies with larger population and more precise measurement are expected to confirm the results and as well examine the clinical application possibility.

## 5. Conclusion

Our findings provide systematic evidence on the modulation of interhemispheric interaction by iTMS with different I-wave selectivity. We first demonstrate that both iTMS with I1- and I3-wave selectivity induce an interhemispheric facilitatory effect. Moreover, iTMS with different I-wave selectivity differs in the modulation of interhemispheric interaction. The discovery from this study also provides a novel clue on the interhemispheric facilitation in the human motor cortex, which might be relevant to the improvement for clinical neurological treatments.

## Data availability statement

The data presented in this study are available on request from the corresponding author.

## Ethics statement

The studies involving human participants were reviewed and approved by the Ethics Committee at Tohoku University Hospital (Protocol Identification Number: 25767). The patients/participants provided their written informed consent to participate in this study.

## Author contributions

DT: conceptualization, methodology, software, validation, formal analysis, experiment conduction, data curation, writing—original draft, and visualization. S-II: conceptualization, methodology, validation, writing—review and editing, supervision, and project administration. All authors contributed to the article and approved the submitted version.

## Funding

This work was supported by JST SPRING, grant number JPMJSP2114, to DT.

## Conflict of interest

The authors declare that the research was conducted in the absence of any commercial or financial relationships that could be construed as a potential conflict of interest.

## Publisher’s note

All claims expressed in this article are solely those of the authors and do not necessarily represent those of their affiliated organizations, or those of the publisher, the editors and the reviewers. Any product that may be evaluated in this article, or claim that may be made by its manufacturer, is not guaranteed or endorsed by the publisher.
